# One-pot synthesis of CoFe_2_O_4_/rGO hybrid hydrogels with 3D networks for high capacity electrochemical energy storage devices

**DOI:** 10.1039/c8ra00285a

**Published:** 2018-02-26

**Authors:** Lingxia Zheng, Lingtong Guan, Guang Yang, Sanming Chen, Huajun Zheng

**Affiliations:** Department of Applied Chemistry, Zhejiang University of Technology Hangzhou 310032 China zhenghj@zjut.edu.cn; State Key Laboratory Breeding Base of Green Chemistry Synthesis Technology, Zhejiang University of Technology Hangzhou 310032 China; College of Chemistry and Environmental Engineering, Shenzhen University Shenzhen 518060 China

## Abstract

CoFe_2_O_4_/reduced graphene oxide (CoFe_2_O_4_/rGO) hydrogel was synthesized *in situ via* a facile one-pot solvothermal approach. The three-dimensional (3D) network structure consists of well-dispersed CoFe_2_O_4_ nanoparticles on the surfaces of graphene sheets. As a binder-free electrode material for supercapacitors, the electrochemical properties of the CoFe_2_O_4_/rGO hybrid hydrogel can be easily adjusted by changing the concentration of the graphene oxide (GO) precursor solution. The results indicate that the hybrid material made using 3.5 mg mL^−1^ GO solution exhibits an outstanding specific capacitance of 356 F g^−1^ at 0.5 A g^−1^, 68% higher than the pure CoFe_2_O_4_ counterpart (111 F g^−1^ at 0.5 A g^−1^), owing to the large specific surface area and good electric conductivity. Additionally, an electrochemical energy storage device based on CoFe_2_O_4_/rGO and rGO was assembled, which exhibits a high energy density of 17.84 W h kg^−1^ at a power density of 650 W kg^−1^ and an excellent cycling stability with 87% capacitance retention at 5 A g^−1^ after 4000 cycles. This work takes one step further towards the development of 3D hybrid hydrogel supercapacitors and highlights their potential application in energy storage devices.

## Abstract 2

CoFe_2_O_4_/reduced graphene oxide (CoFe_2_O_4_/rGO) hydrogel was synthesized *in situ via* a facile one-pot solvothermal approach.

## Graphical abstract

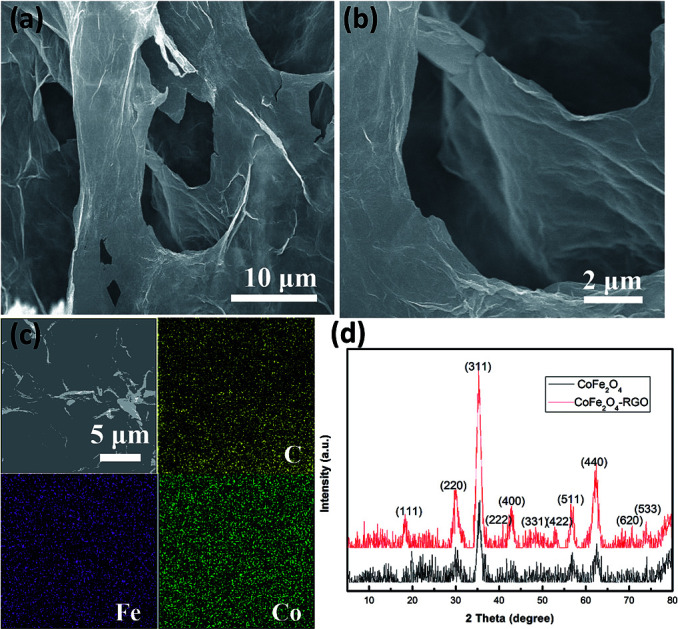

## Introduction

In recent years, the conversion and storage of energy originating from green and renewable resources has becoming increasingly in demand. Supercapacitors (also known as electrochemical capacitors), as new energy storage systems, have attracted huge attention due to their fast charge/discharge rate, superior power density and long cycle life.^1–6^ However, the low energy density (normally less than 10 W h kg^−1^) has limited their commercial applications.^1,6^ In the past few years, numerous researchers have already made many efforts to improve the specific capacitance by developing nanostructured electrode materials or increasing the operating voltage of supercapacitors without sacrificing their power density and cycle life.^7–11^

The electrochemical performance of supercapacitors depends mainly on the structure and properties of the electrode materials. To date, various materials such as transition metal oxides, metal hydroxides and carbon-based materials have been extensively explored.^12–19^ Among them, one kind of binary metal oxide, CoFe_2_O_4_, has been widely used as an electrode material for lithium ion batteries and supercapacitors, due to its high theoretical specific capacitance, good chemical stability, and good electrical and magnetic properties.^20–23^ However, the practical application of pure CoFe_2_O_4_ is limited because of its poor electrical conductivity. To address this problem, many efforts have been devoted to developing nanocomposites based on CoFe_2_O_4_ and carbon materials.^24–29^ For instance, a ternary cobalt ferrite/graphene/polyaniline composite was prepared *via* a combination of hydrothermal and polymerization processes, and was found to exhibit a high specific capacitance of 1133.3 F g^−1^, superior rate capability and excellent cycling stability.^24^ Similarly, He *et al.* reported that the specific capacitance and cycling stability of reduced graphene oxide–CoFe_2_O_4_ composites synthesized using a co-precipitation method were greatly improved compared with those of the pure CoFe_2_O_4_ electrode.^30^ Such composite materials not only can improve the specific capacitance, but also contribute to an improved rate capability. Thus, it is advantageous to introduce carbonaceous species into nanostructured CoFe_2_O_4_-based electrode materials to enhance their electrochemical properties.

Graphene, a typical two-dimensional (2D) one-atom-thick carbon material, has attracted considerable attention because of its appealing features including huge surface area, superb thermal conductivity, electronic conductivity, and mechanical properties.^31–35^ Interestingly, Shi's group recently reported a self-assembled graphene hydrogel in a 3D network *via* a convenient one-step hydrothermal method, which exhibited good electrical conductivity, mechanical strength and thermal stability, showing promise for high performance supercapacitors.^36^ Many researchers have carried out intensive studies on various electrode materials based on graphene hydrogels.^37–42^ For example, Wang *et al.* reported that NiOOH nanosheet/graphene hydrogel-based electrode materials, obtained *via* combined solvothermal and hydrothermal reactions, delivered a high capacitance of 1162 F g^−1^ at 1 A g^−1^ and 981 F g^−1^ at 20 A g^−1^.^38^ Zhu *et al.* assembled a hierarchical and interconnected reduced graphene oxide/β-MnO_2_ nanobelt hybrid hydrogel *via* a hydrothermal route, which exhibited higher specific capacitance and better cycling stability than pure β-MnO_2_ nanobelts.^39^ Nevertheless, the synthesis of CoFe_2_O_4_ nanostructures with a self-assembled 3D graphene hydrogel composite for use in supercapacitors has rarely been reported.

Herein, novel CoFe_2_O_4_ nanoparticles/reduced graphene oxide (CoFe_2_O_4_/rGO) hybrid hydrogels were synthesized *via* a facile one-pot solvothermal method. The attachment of uniformly-distributed CoFe_2_O_4_ nanoparticles on the surfaces of graphene sheets is beneficial for providing more electroactive sites for faradic redox reactions. Interestingly, the CoFe_2_O_4_/rGO hybrid hydrogels possess a 3D mesoporous network, which can offer easy diffusion of the electrolyte and efficient pathways for electron transfer and ion transport. As binder-free electrode materials for supercapacitors, the CoFe_2_O_4_/rGO hybrid hydrogels exhibit excellent electrochemical performance with high capacitance, good rate capability and remarkable cycling stability. In addition, an electrochemical energy storage device was assembled, using CoFe_2_O_4_/rGO hybrid hydrogel as the positive electrode and rGO hydrogel as the negative electrode, and was found to deliver high energy density and cycling stability. The charming electrochemical properties demonstrate the potential application of CoFe_2_O_4_/rGO hybrid hydrogels in high performance supercapacitors.

## Experimental section

### One-pot solvothermal synthesis of CoFe_2_O_4_/rGO hybrid hydrogels

Graphite powder (400 mesh) was purchased from Port Co., Ltd. (Shenzhen, China). KMnO_4_, NaNO_3_, HCl (37 wt%), H_2_SO_4_ (98%) and H_2_O_2_ (30%) were purchased from Aladdin Industrial (Shanghai, China). All the reagents were of analytical grade and used directly without further purification in the experiments.

GO was prepared according to a modified Hummers' method.^40–43^ The CoFe_2_O_4_/rGO hybrid hydrogel was prepared using a solvothermal method. In a typical procedure, GO powder was dispersed in 30 mL of deionized water to form a homogeneous aqueous solution (2 mg mL^−1^). 1 mmol of Co(NO_3_)_2_·6H_2_O, 2 mmol of Fe(NO_3_)_2_·6H_2_O and 5 mmol of sodium acetate were dissolved into 30 mL of ethylene glycol. Then the above solutions were mixed together and vigorously stirred for 1 h. Subsequently, the mixed solution was transferred into an 80 mL Teflon-lined stainless-steel autoclave and kept at 180 °C for 12 h. After cooling down to room temperature naturally, the CoFe_2_O_4_/rGO hydrogels were rinsed with deionized water several times. For further characterization, freeze-drying technology under vacuum was employed to obtain aerogels. Four different CoFe_2_O_4_/rGO hybrid hydrogels were prepared by changing the concentration of the GO precursor solution (2, 3, 3.5 and 4 mg mL^−1^). For comparison, a pure CoFe_2_O_4_ sample was fabricated by following the same solvothermal process in the absence of GO. Meanwhile, a pure rGO hydrogel was prepared by using the same solvothermal process without inorganic metal salts as precursors.

### Structural characterization

The morphology and microstructure of these samples were characterized using scanning electron microscopy (SEM, JEOL Hitachi S-4700, Japan) and high-resolution transmission electron microscopy (HRTEM, JEOL JEM200CX, JEOL). Powder X-ray diffraction (XRD, Bruker D8 Advance diffractometer with Cu-Kα radiation) experiments were performed to study the crystallographic information of the samples. X-ray photoelectron spectroscopy (XPS, Thermo Fisher Scientific, USA) was performed using an ESCALab MKII spectrometer with Al Kα (1.4866 keV) as the X-ray source. The specific Brunauer–Emmett–Teller (BET) surface areas of the hydrogels were measured by analyzing the nitrogen adsorption and desorption isotherms at −196 °C, obtained using a Micromeritics Model ASAP 2020 sorptometer.

### Electrochemical measurements

Electrochemical properties were measured on an IVIUM electrochemical workstation in 6 M KOH solution as the electrolyte. A three-electrode system was employed, where Pt and Hg/HgO were used as the counter electrode and the reference electrode, respectively. The pure CoFe_2_O_4_ electrode was prepared by mixing 80 wt% CoFe_2_O_4_ powder with 10 wt% acetylene black and 10 wt% polytetrafluorene ethylene (PTFE) binder together. Then, drops of isopropyl alcohol were used to form slurries thoroughly. After drying in a vacuum oven at 60 °C for 8 h, the mixture was pressed onto nickel foam current collectors (1 × 1 cm^2^). The CoFe_2_O_4_/rGO hybrid hydrogel electrode was prepared by cutting a piece of freeze-dried hydrogel, and then the patch was pressed onto the nickel foam current collectors (1 × 1 cm^2^). Cyclic voltammetry (CV) and galvanostatic charge/discharge (GCD) measurements were carried out to test the electrochemical properties of the as-prepared electrodes. Electrochemical impedance spectroscopy (EIS) was preformed over a frequency range from 10^5^ to 0.01 Hz at an amplitude of 5 mV. The capacitive performance of the electrodes in a two-electrode configuration was also assessed using CV and GCD, and a LAND battery system was used to examine the cycling stability. A battery-capacitor hybrid device was fabricated using CoFe_2_O_4_/rGO hybrid hydrogel as the positive electrode and rGO hydrogel as the negative electrode. The capability of the hybrid devices (CoFe_2_O_4_/rGO//rGO) (two single devices were connected in series) was evaluated by powering a light-emitting-diode (LED 3 V, 0.06 W).

## Results and discussion

### Structural characterization

**Fig. 1:**
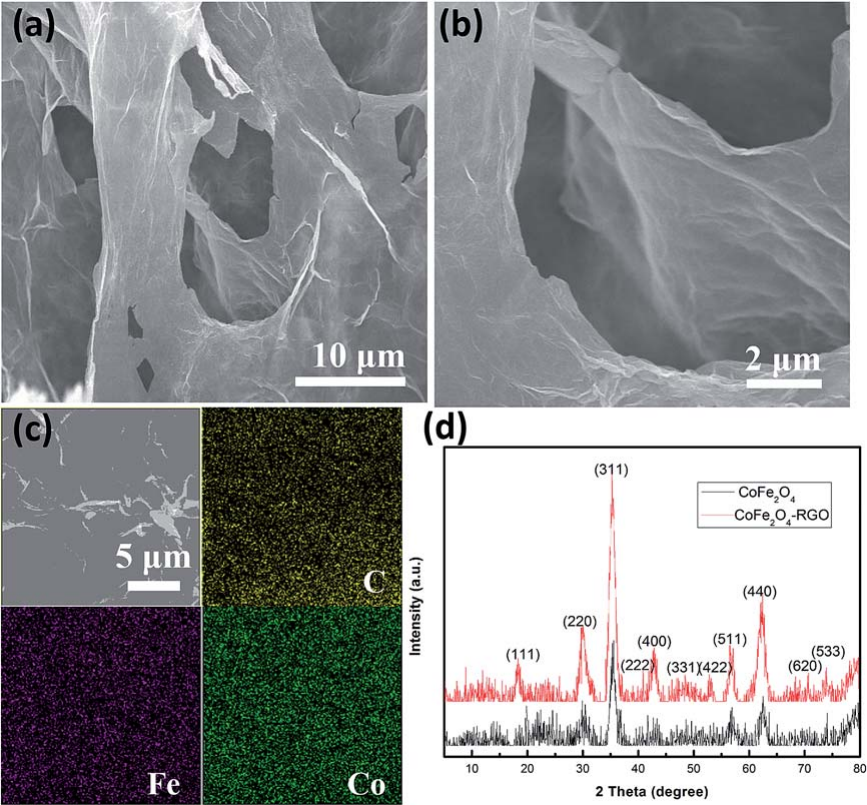
(a and b) SEM images, and (c) elemental mapping of the CoFe_2_O_4_/rGO hybrid hydrogel. (d) XRD patterns of the pure CoFe_2_O_4_ and CoFe_2_O_4_/rGO hybrid hydrogel.

The morphology and crystalline structure of the CoFe_2_O_4_/rGO hybrid hydrogel were investigated by SEM and XRD. Fig. 1a and b show the SEM images of the CoFe_2_O_4_/rGO hybrid hydrogel after freeze-drying treatment under vacuum, displaying a 3D porous network with pore sizes ranging from sub-micrometers to several micrometers. This network is beneficial for enhancing the electrochemical properties, as it not only provides an efficient pathway for electron transport but also reduces the ion diffusion resistance of the electrolyte for charge storage reactions.^36,38^ In addition, the 3D porous network provides abundant active sites to load CoFe_2_O_4_ nanoparticles. As shown in the elemental mapping images (Fig. 1c), C, Fe and Co are uniformly distributed in the composite, suggesting that the CoFe_2_O_4_ nanoparticles are homogeneously dispersed on the surface of the graphene sheets. Fig. 1d depicts the XRD patterns of the pure CoFe_2_O_4_ and the CoFe_2_O_4_/rGO hybrid, and the characteristic diffraction peaks at 18.3°, 30.1°, 35.4°, 37.1°, 43.1°, 53.4°, 57.0°, 62.6° and 74.0° can be assigned to the (111), (220), (311), (222), (400), (422), (511), (440) and (533) crystal planes of cubic spinel CoFe_2_O_4_ (JCPDS no. 22-1086), respectively.^44^ After forming a composite with rGO, the highly crystalline nature of the CoFe_2_O_4_/rGO hybrid sample did not change. However, the conventional graphene-sheet stacking peak at 2*θ* = 24.5° assigned to the (002) plane cannot be obviously seen, indicating that the graphene sheets might be restacked during the reduction process, resulting in the amorphous nature.^26^

**Fig. 2:**
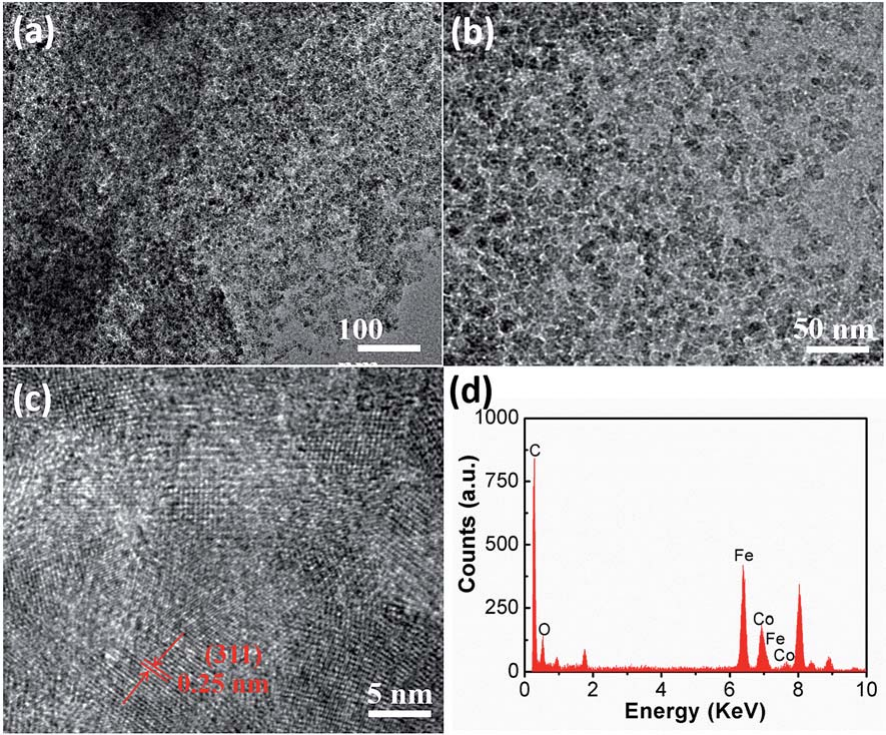
(a and b) TEM images, (c) HR-TEM image and (d) EDX spectrum of the CoFe_2_O_4_/rGO hybrid hydrogel.

To further investigate the details of the morphology and composition, TEM, HRTEM and EDX were employed. The TEM images (Fig. 2a and b) confirm that the CoFe_2_O_4_ nanoparticles are well-dispersed and firmly anchored on the graphene sheets. The HRTEM image in Fig. 2c reveals that the clear lattice fringe with an interplanar spacing of 0.25 nm corresponds well to the (311) plane of CoFe_2_O_4_. The EDX results (Fig. 2d) show the elements Co, Fe, O and C, which are in accordance with the nanocomposite and a Co/Fe atom ratio of about 1 : 2, further demonstrating the successful synthesis of the CoFe_2_O_4_/rGO hybrid hydrogel.

**Fig. 3:**
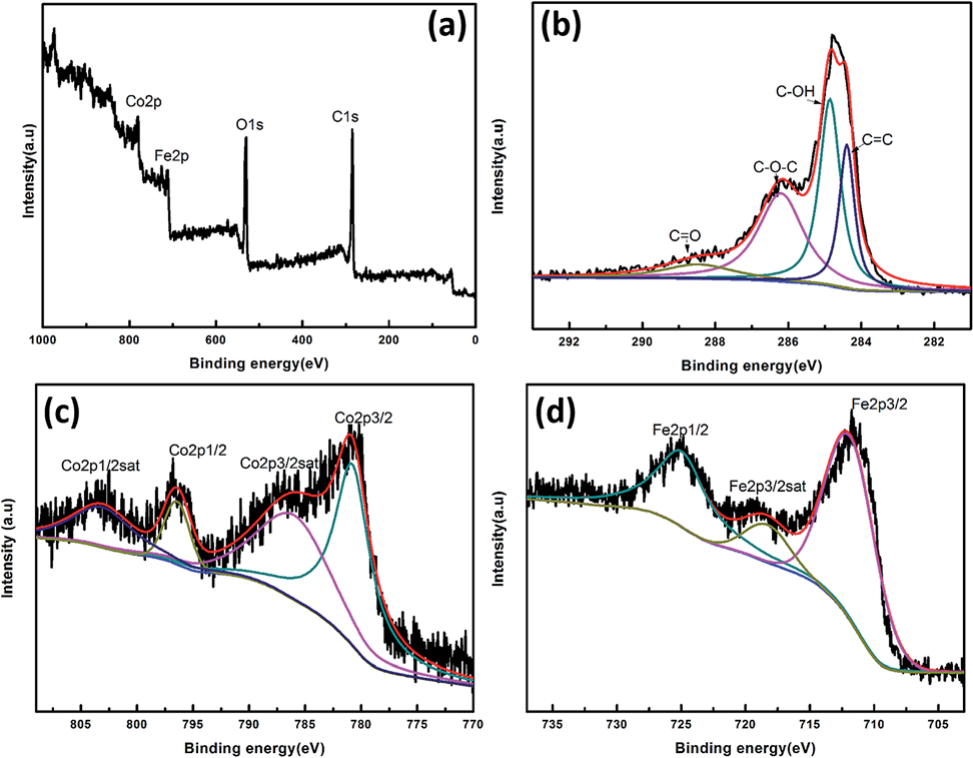
XPS spectra of CoFe_2_O_4_/rGO composite: (a) survey scan, (b) C 1s, (c) Co 2p, and (d) Fe 2p spectrum.

The chemical structure and valence states were studied using X-ray photoelectron spectroscopy (XPS) as shown in Fig. 3. The survey scan XPS spectrum of the CoFe_2_O_4_/rGO composite shows the existence of the elements Fe, Co, C and O. The high-resolution C1 XPS spectrum in Fig. 3b demonstrates plenty of functional groups on the graphene sheets. For example, the C 1s peaks at 284.7 and 286.5 eV can be attributed to the C=C and C–O groups of rGO in the composite, respectively.^42^ The high resolution Co 2p spectrum (Fig. 3c) is deconvoluted into two spin–orbit doublets located at 781.6 and 796.7 eV, corresponding to the electronic states of Co 2p_3/2_ and Co 2p_1/2_, respectively, accompanied by two shakeup satellites centered at 784.8 and 803.8 eV. The main spin-energy separation of 15.1 eV is a signature of the Co^2+^ oxidation state.^45^ As shown in Fig. 3d, in addition to the satellite peak at 716.7 eV, the peaks at 711.8 and 725.6 eV correspond to Fe 2p_3/2_ and Fe 2p_1/2_ spin–orbit peaks, respectively, which are attributed to the dominant states of Fe^3+^.^44,46,47^ The 2p_3/2_–2p_1/2_ separation and satellite structures feature a broad peak in the sample, which is characteristic of high-spin octahedral cations, indicating the existence of some Fe^2+^ components.^48^ As indicated by XPS analysis, CoFe_2_O_4_/rGO hybrid hydrogels were successfully synthesized.

**Fig. 4:**
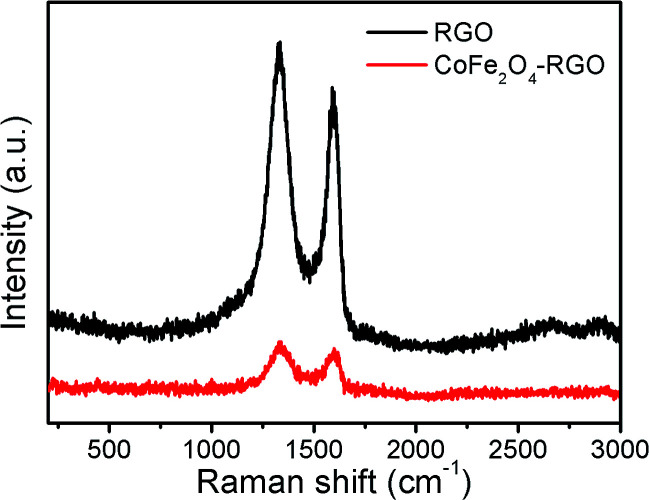
Raman spectra of rGO and the CoFe_2_O_4_/rGO hybrid hydrogel.

Fig. 4 shows the Raman spectra of rGO and the CoFe_2_O_4_/rGO hybrid hydrogel. Both display a D-band (∼1350 cm^−1^) related to the breathing mode of sp^2^ carbon atoms and activated by the existence of oxygen-containing groups,^49^ and a G-band (∼1590 cm^−1^) ascribed to the edge planes and disordered structure. Interestingly, the 2D-band is missing in both samples, possibly because the rGO hydrogel processes a network structure instead of a monolayer graphene structure.^50^ The intensity ratio of D-band to G-band (*I*_D_/*I*_G_ = *ca.* 1.2) indicates that several functional groups still exist in the graphene in the pure rGO sample,^51^ which is beneficial for composite formation with CoFe_2_O_4_. Unfortunately, due to the large amount of rGO in the hybrid hydrogel sample, the spinel CoFe_2_O_4_ phase cannot been clearly seen.

**Fig. 5:**
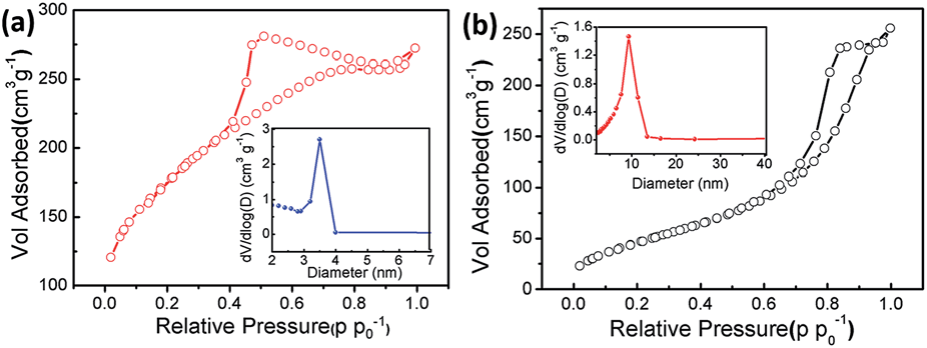
Nitrogen adsorption and desorption isotherms of freeze-dried CoFe_2_O_4_/rGO composite (a) and pure CoFe_2_O_4_ sample (b). The insets show their corresponding pore size distributions.

Nitrogen physisorption measurements were conducted to characterize the porous features of the pure CoFe_2_O_4_ and CoFe_2_O_4_/rGO composite. As presented in Fig. 5, the specific BET surface area of the CoFe_2_O_4_/rGO composite was calculated to be 614.4 m^2^ g^−1^, while that of the pure CoFe_2_O_4_ nanoparticle sample is only 179.7 m^2^ g^−1^. This indicates that after the reaction with the rGO hydrogel, the obtained CoFe_2_O_4_/rGO composite maintains a 3D structure with a remarkably increased surface area, which is 3.4 times larger than that of pure CoFe_2_O_4_, suggesting that the construction of a 3D framework *via* a hydrothermal route is an effective way to achieve nanocomposites with a high surface area. As displayed in Fig. 5a, the CoFe_2_O_4_/rGO composite exhibits a typical type-IV hysteresis loop at a relative pressure of between 0.4 and 0.9, and the pore size distribution is centered at 3.5 nm (inset in Fig. 5a), while the pore size distribution for the pure CoFe_2_O_4_ sample is mainly located at ∼10 nm (inset in Fig. 5b). The higher surface area and much smaller mesopores of the CoFe_2_O_4_/rGO composite would provide a more convenient channel for ion diffusion and electron transfer, leading to a higher electrochemical capacity. Moreover, the introduction of the rGO hydrogel not only reduces the agglomeration of CoFe_2_O_4_ nanoparticles, but the attachment of CoFe_2_O_4_ nanoparticles can also weaken the strong interaction between the rGO sheets, which is beneficial for the formation of the porous rGO-based aerogel.^42^

### Electrochemical characterization

**Fig. 6:**
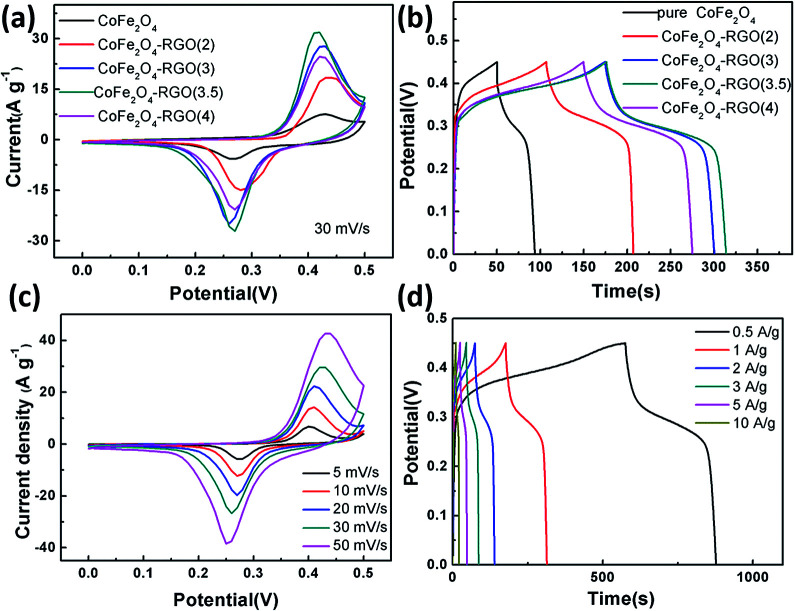
(a) CV curves at a scan rate of 30 mV s^−1^, and (b) GCD curves at a current density of 1 A g^−1^ for the different electrodes. (c) CV curves at different scan rates, and (d) GCD curves at different current densities ranging from 0.5 to 10 A g^−1^ for the CoFe_2_O_4_–rGO(3.5) sample.

The electrochemical properties of the pure CoFe_2_O_4_ nanoparticle sample and the four CoFe_2_O_4_/rGO hybrid hydrogels with different GO precursor solution concentrations were firstly evaluated in a three-electrode cell, and the results are shown in Fig. 6. The cyclic voltammetry (CV) curves in Fig. 6a were measured at a sweep rate of 30 mV s^−1^ within the potential window of 0–0.5 V in 6 M KOH aqueous solution. A pair of symmetric redox peaks can be clearly observed on each CV curve, indicating that the capacitance characteristics are mainly governed by surface faradaic redox mechanisms.^20^ The reaction of the conversion in the electrolyte between different cobalt and iron oxidation states can be described by the following equation:

1 CoFe_2_O_4_ + OH^−^ + H_2_O ↔ CoOOH + FeOOH + e^−^

Furthermore, the area of the CV curves for the CoFe_2_O_4_/rGO composite synthesized with a GO precursor solution concentration of 3.5 mg mL^−1^ (denoted as CoFe_2_O_4_/rGO(3.5)) is larger than that of the other three hybrid samples, showing the highest specific capacitance. They follow the order: CoFe_2_O_4_/rGO(3.5) > CoFe_2_O_4_/rGO(3) > CoFe_2_O_4_/rGO(4) > CoFe_2_O_4_/rGO(2). Galvanostatic charge–discharge (GCD) measurements at a current density of 1 A g^−1^ within a potential window of 0–0.5 V (*vs.* SCE) were conducted to further estimate the best ratio of CoFe_2_O_4_ and rGO (Fig. 6b). A distinct potential plateau region and nearly symmetric curves for all of the electrodes are observed, suggesting the superb coulombic efficiency of the charge–discharge process. The specific capacitance of the different hybrid hydrogel samples can be calculated according to eqn (2):2
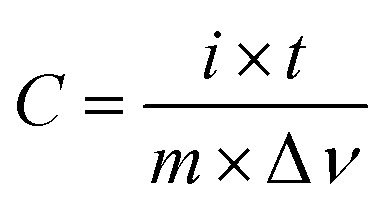
where *C* is the specific capacitance, *i* is the discharge current, *t* is discharge time, *m* is the mass of the active materials and Δ*ν* is the potential window. The specific capacitance values of those curves are 99.8 (CoFe_2_O_4_), 162.2 (CoFe_2_O_4_/rGO(2)), 284 (CoFe_2_O_4_/rGO(3)), 310.7 (CoFe_2_O_4_/rGO(3.5)) and 271.2 F g^−1^ (CoFe_2_O_4_/rGO(4)). It is noteworthy that the hybrid sample CoFe_2_O_4_/rGO(3.5) delivers the largest specific capacitance, which is in accordance with the area results from the CV curves in Fig. 6a. To further explore the electrochemical performance of the hybrid hydrogels, the CoFe_2_O_4_/rGO(3.5) sample was examined using CV at different scan rates and GCD at different current densities. Similarly, the CV curves measured at scan rates from 5 to 50 mV s^−1^, as shown in Fig. 6c, exhibit a pair of symmetric redox peaks on each CV curve and the shapes are slightly different. The oxidation peak shifts to a higher potential, while the reduction peak shifts to a lower potential as the scan rate increases. A high symmetry at different current densities from 0.5 to 10 A g^−1^ is displayed in the GCD curves for the CoFe_2_O_4_/rGO(3.5) electrode (Fig. 6d). The specific capacitance values were calculated to be 356, 310.7, 298, 280, 265 and 244 F g^−1^ at current densities of 0.5, 1, 2, 3, 5 and 10 A g^−1^, respectively. The hybrid hydrogel electrode maintains capacitance retention as high as 68.5% after increasing the current density 20-fold, indicating good rate capability.

**Fig. 7:**
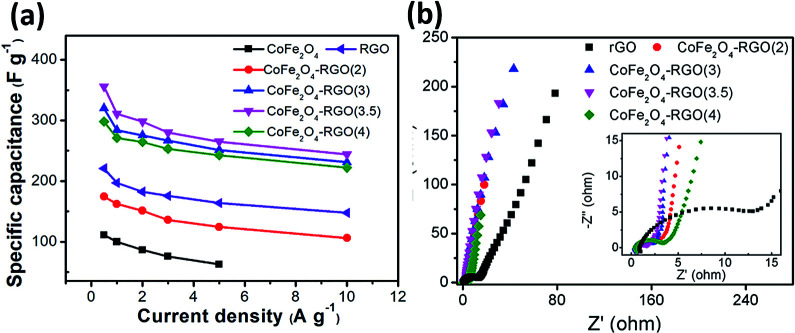
(a) Comparison of specific capacitance at different current densities and (b) Nyquist plots for different electrodes.

The comparison of the specific capacitance for different composite samples with various current densities is plotted in Fig. 7a. With increasing current density, the specific capacitance for all the electrodes decreases gradually. This phenomenon is possibly attributed to the insufficient electroactive material involved in the faradaic reactions at higher current densities. These samples follow the trend: CoFe_2_O_4_/rGO(3.5) > CoFe_2_O_4_/rGO(3) > CoFe_2_O_4_/rGO(4) > rGO > CoFe_2_O_4_/rGO(2) > CoFe_2_O_4_, which is in good agreement with the results in Fig. 6a and b. This indicates that the introduction of an appropriate amount of rGO into CoFe_2_O_4_ nanoparticles is helpful for enhancing the specific capacitance. Meanwhile, varying the loading amount of the excellent conductive material rGO greatly affects the electrical conductivity of the composites, as indicated in the EIS spectra in Fig. 7b. The Nyquist plots for all the electrode samples display a depressed semicircle in the high-frequency region, corresponding to the charge transfer resistance, and a straight line in the low-frequency region, reflecting the diffusion of the electroactive species. The CoFe_2_O_4_/rGO(3.5) electrode demonstrates the smallest semicircles, suggesting that it has the smallest charge transfer resistance, thus leading to the largest specific capacitance.

To further investigate the electrochemical properties of the CoFe_2_O_4_/rGO hybrid hydrogel electrodes in a full-cell configuration, an electrochemical energy storage device was assembled using the optimal CoFe_2_O_4_/rGO(3.5) sample as the positive electrode and the reduced graphene oxide (rGO) hydrogel as the negative electrode. To balance the charge storage between the positive electrode and negative electrode, the masses of the two electrodes were calculated according to eqn (3):3
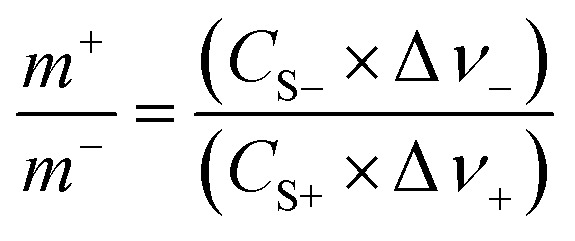
where *m*^+^ and *m*^−^ are the masses of the positive and negative electrodes (g), respectively, and the specific capacitance *C*_S−_, *C*_S+_ (F g^−1^), and the voltage range Δ*ν*_−_, Δ*ν*_+_ (V) are for the negative and positive electrodes, respectively.^39^

**Fig. 8:**
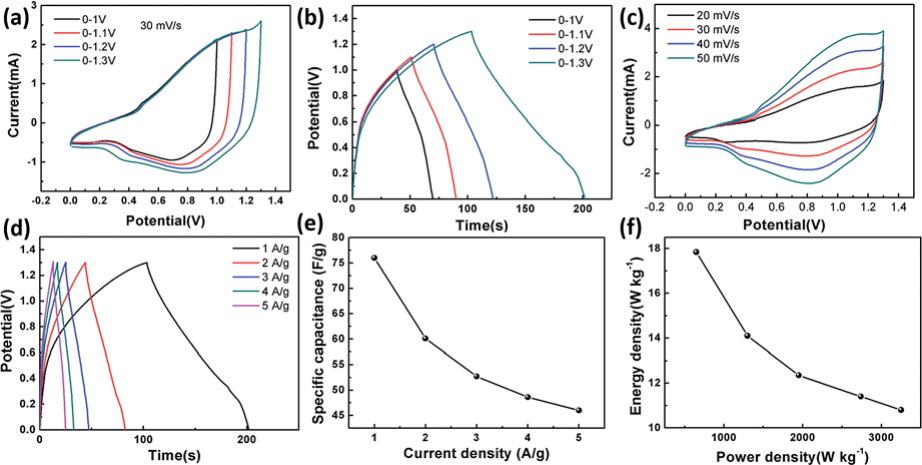
The electrochemical properties of the CoFe_2_O_4_/rGO//rGO hybrid device in a two-electrode cell. (a) CV curves at different potential windows. (b) GCD curves at a current density of 1 A g^−1^. (c) CV curves at different scan rates. (d) GCD curves at different current densities. (e) Specific capacitance at different current densities. (f) Ragone plots.

The specific capacitance of CoFe_2_O_4_/rGO(3.5) is 310.7 F g^−1^, while that of rGO is 197.5 F g^−1^ at a current density of 1 A g^−1^ (Fig. 7a). The potential windows of the CoFe_2_O_4_/rGO hybrid electrode and the pure rGO electrode are 0–0.5 V and −0.8–0 V, respectively. Therefore, the mass ratio 
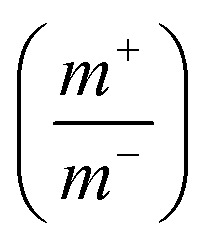
 between the two electrodes is 1.02. Fig. 8a displays the CoFe_2_O_4_/rGO//rGO electrochemical energy storage device in 6 M KOH solution with different potential windows varying from 0–1 V to 0–1.3 V at a scan rate of 30 mV s^−1^. When the cell voltage reaches 1.3 V, there is no obvious change of anodic current, indicating that the cell electrolyte is stable at 1.3 V. The GCD curves of the hybrid device under different voltage ranges at 1 A g^−1^ are revealed in Fig. 8b. The curves of each GCD exhibit a good symmetrical shape. In particular, the potential window of the hybrid device is beyond the thermodynamic limit of ∼1.2 V and thus greatly improves the energy density.^44,48,52^ The potential window of 0–1.3 V was chosen for further investigation with CV and GCD tests. Fig. 8c shows the CV curves of the CoFe_2_O_4_/rGO//rGO hybrid device at scan rates varying from 20 to 50 mV s^−1^. With increasing scan rate, the peak current increases. Moreover, the CV curves show two relatively weak redox peaks, revealing synergistic contributions from both the electric double-layer capacitance and the pseudocapacitance. The specific capacitances calculated from the GCD curves in Fig. 8d of the hybrid device are 76, 60.1, 52.7, 48.6 and 46 F g^−1^ at a current density of 1, 2, 3, 4 and 5 A g^−1^, respectively (Fig. 8e). The energy density and power density were calculated according to eqn (4) and (5), respectively:4
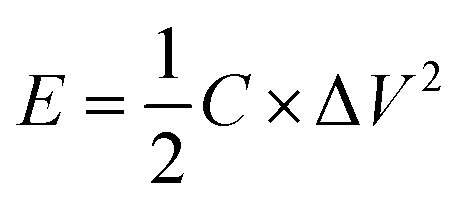
5
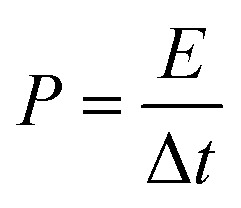
where *C* is the specific capacitance, Δ*V* is the potential window, and Δ*t* is the discharge time. The hybrid device delivers a maximum energy density of 17.84 W h kg^−1^ at a power density of 650 W kg^−1^ and a maximum power density of 3250 W kg^−1^ at an energy density of 10.8 W h kg^−1^ (Fig. 8f).

**Fig. 9:**
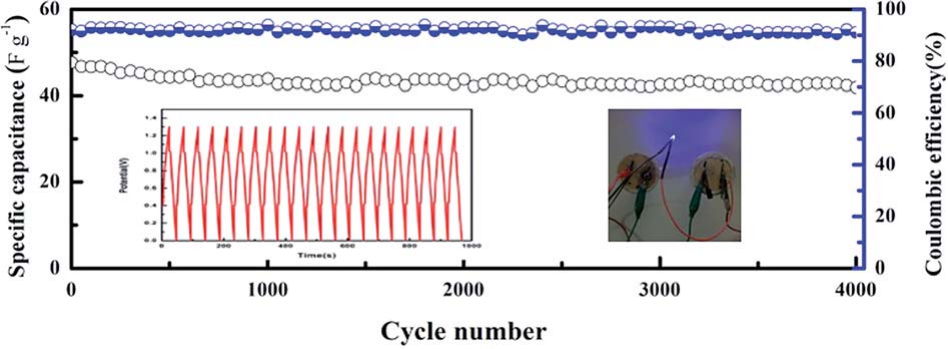
Cycling performance and coulombic efficiency at 5 A g^−1^. The inset on the left shows the GCD curves for the first 20 cycles, while the inset on the right shows the photograph of an LED powered by two CoFe_2_O_4_/rGO//rGO hybrid devices connected in series.

The cycling performance of the CoFe_2_O_4_/rGO//rGO hybrid device was also tested for charge–discharge cycles at a high current density of 5 A g^−1^, and the specific capacitance was found to still be stable at 40.2 F g^−1^ with 87% retention of the initial value after 4000 cycles (Fig. 9). Additionally, the coulombic efficiency remains near 92% after 4000 cycles, further confirming the excellent cycling stability and reversibility of the faradic reactions. Table 1 lists the main electrochemical parameters of the CoFe_2_O_4_/rGO hybrid hydrogel electrode compared with the related electrode materials in the literature. It is obvious that the specific capacitance of the novel hydrogel electrode material is comparable with the best values obtained for related CoFe_2_O_4_-based electrode materials that possess excellent stability. Moreover, two CoFe_2_O_4_/rGO//rGO devices connected in series can power a light emitting diode (LED) with a voltage of 3.0 V for several seconds (inset (right) in Fig. 9), confirming their promising application in energy storage devices.

**Table 1:** Comparison of the electrochemical performances of CoFe_2_O_4_/rGO hybrid hydrogels with other related electrode materials in the literature

Electrode materials	Methods	Specific capacitance	Capacity retention	Ref.
CoFe_2_O_4_/rGO	Co-precipitation	123.2 F g^−1^ (5 mA cm^−2^)	78.1% (1000 cycles)	30
CoFe_2_O_4_/graphene	Hydrothermal	166.5 C g^−1^ (0.5 A g^−1^)	79.3% (5000 cycles)	48
FeMnO_3_/graphene	Thermal treatment	189 F g^−1^ (0.5 A g^−1^)	75.4% (1500 cycles)	52
NiCo_2_O_4_/CNTs	Thermal treatment	210 F g^−1^ (2 A g^−1^)	92.7% (2500 cycles)	53
CoFe_2_O_4_/rGO	Solution combustion	195 F g^−1^ (1 mV s^−1^)	66.7% (3000 cycles)	54
MnFe_2_O_4_/carbon black/polyaniline	Hydrothermal & polymerization	205 F g^−1^ (0.5 A g^−1^)	80% (10 000 cycles)	55
CoFe_2_O_4_/rGO/polyaniline	Solution combustion & polymerization	257 F g^−1^ (1 mV s^−1^)	—	56
NiFe_2_O_4_/rGO	Hydrothermal	345 F g^−1^ (1 A g^−1^)	60% (300 cycles)	57
**CoFe**_**2**_ **O**_**4**_ **/rGO hydrogel**	**Hydrothermal**	**356 F g** ^**−1**^ **(0.5 A g** ^**−1**^ **)**	**87% (4000 cycles)**	**This work**

## Conclusions

In summary, we have presented a scalable strategy to construct a unique architecture comprising 3D porous rGO hydrogel-supported CoFe_2_O_4_ nanoparticles, through a facile one-pot solvothermal process. The as-prepared CoFe_2_O_4_ nanoparticles are evenly distributed on the surfaces of graphene sheets. When used as a binder-free supercapacitor electrode material, CoFe_2_O_4_/rGO synthesized using 3.5 mg mL^−1^ GO precursor solution shows a high capacitance of up to 356 F g^−1^ at 0.5 A g^−1^, which is 3.2 times higher than the pure CoFe_2_O_4_ nanoparticle electrode, due to the large specific surface area and excellent electric conductivity. Moreover, the CoFe_2_O_4_/rGO//rGO electrochemical energy storage device exhibits a high energy density of 17.84 W h kg^−1^ at a power density of 650 W kg^−1^ and 87% capacitance retention at 5 A g^−1^ after 4000 cycles. The excellent electrochemical performance indicates that CoFe_2_O_4_/rGO hybrid hydrogels hold great promise for high-performance energy storage devices.

## Conflicts of interest

There are no conflicts to declare.

## Supplementary Material
